# Self-reported symptoms of arm lymphedema and health-related quality of life among female breast cancer survivors

**DOI:** 10.1038/s41598-021-89055-0

**Published:** 2021-05-21

**Authors:** Kayo Togawa, Huiyan Ma, Ashley Wilder Smith, Marian L. Neuhouser, Stephanie M. George, Kathy B. Baumgartner, Anne McTiernan, Richard Baumgartner, Rachel M. Ballard, Leslie Bernstein

**Affiliations:** 1 Department of Population Sciences, Beckman Research Institute, City of Hope, Duarte, CA USA; 2 Environment and Lifestyle Epidemiology Branch, International Agency for Research on Cancer (IARC/WHO), Lyon, France; 3 Division of Cancer Control and Population Sciences, National Cancer Institute, Bethesda, MD USA; 4 Division of Public Health Sciences, Fred Hutchinson Cancer Research Center, Seattle, WA USA; 5 Office of Disease Prevention, Office of the Director, National Institutes of Health, Bethesda, MD USA; 6 National Institute for Arthritis, Musculoskeletal and Skin Diseases, National Institutes of Health, Bethesda, MD USA; 7 Department of Epidemiology and Population Health, James Graham Brown Cancer Center, University of Louisville, Louisville, KY USA

**Keywords:** Outcomes research, Breast cancer, Epidemiology, Oedema

## Abstract

We examined cross-sectional associations between arm lymphedema symptoms and health-related quality of life (HRQoL) in the Health, Eating, Activity and Lifestyle (HEAL) Study. 499 women diagnosed with localized or regional breast cancer at ages 35–64 years completed a survey, on average 40 months after diagnosis, querying presence of lymphedema, nine lymphedema-related symptoms, e.g., tension, burning pain, mobility loss, and warmth/redness, and HRQoL. Analysis of covariance models were used to assess HRQoL scores in relation to presence of lymphedema and lymphedema-related symptoms. Lymphedema was self-reported by 137 women, of whom 98 were experiencing lymphedema at the time of the assessment. The most common symptoms were heaviness (52%), numbness (47%), and tightness (45%). Perceived physical health was worse for women reporting past or current lymphedema than those reporting no lymphedema (*P*-value < 0.0001). No difference was observed for perceived mental health (*P*-value = 0.31). Perceived physical health, stress, and lymphedema-specific HRQoL scores worsened as number of symptoms increased (*P*-values ≤ 0.01). Women reporting tension in the arm had lower physical health (*P*-value = 0.01), and those experiencing burning pain, tension, heaviness, or warmth/redness in the arm had lower lymphedema-specific HRQoL (*P*-values < 0.05). Treatment targeting specific lymphedema-related symptoms in addition to size/volume reduction may improve some aspects of HRQoL among affected women.

## Introduction

More than 3.8 million women are living with a history of invasive breast cancer in the United States^1^. Owing to the improvements in breast cancer diagnosis, therapy and follow-up care, many long-term breast cancer survivors have measured health-related quality of life (HRQoL) similar to that of unaffected women^2–5^; nevertheless, some survivors still encounter specific problems that decrease their HRQoL^3,5^. Arm lymphedema, one of the common problems after breast cancer treatment, has been shown to decrease survivors’ HRQoL^5–10^. Although the improvement in breast cancer treatment has lowered lymphedema incidence, about 6% of women who receive sentinel lymph node biopsy and 20% of women who receive axillary lymph node dissection develop this condition^11^.

Arm lymphedema is generally diagnosed by comparing the at-risk and unaffected arms or pre-surgery and post-surgery measurements and finding either > 2 cm difference in circumference, > 200 ml difference in volume, or > 3–10% difference in circumference or volume^9^. In addition to arm swelling, women with lymphedema may experience physical and psychological symptoms, such as limited arm mobility, pain, sensation of heaviness, numbness in the affected arm, negative self-perception of body image, and emotional distress^9,12,13^. Currently, no cure for lymphedema exists and the condition is usually chronic; thus, many lymphedema-affected women will live with this potentially debilitating condition throughout the rest of their lives^14^.

The common therapies for arm lymphedema include repeated applications of complex physical therapy, manual lymphatic drainage, laser therapy, pneumatic pump therapy, and compression bandaging^15^. While these approaches may effectively control the size or volume of the affected arm, patients may continue to experience other lymphedema symptoms affecting their HRQoL^12^. Most previous studies on lymphedema and HRQoL among breast cancer survivors have focused on presence of arm lymphedema and not specific lymphedema-related symptoms. Therefore, little is known about which lymphedema symptom(s) negatively impact HRQoL. This information is critical for developing interventions that maintain or improve the HRQoL of lymphedema-affected breast cancer survivors.

We, therefore, queried whether breast cancer survivors participating in the longitudinal Health Eating, Activity and Lifestyle (HEAL) Study developed lymphedema and among those with lymphedema, asked whether they experienced specific lymphedema symptoms. At the same time, all women were asked about their HRQoL, which encompasses physical and mental health, stress, fear of recurrence, sexual health, and lymphedema-specific HRQoL. Using this information, we evaluated cross-sectional associations between lymphedema symptoms and HRQoL.

## Materials and methods

### Study setting

Details of the study design and recruitment procedures for the HEAL Study have been published previously^16,17^. Briefly, the HEAL Study is a multi-center, multi-ethnic prospective cohort study that recruited and followed 1,183 female breast cancer survivors. Women diagnosed with first primary in situ or Stage I-IIIA invasive breast cancer between 1995 and 1999 were identified through Surveillance, Epidemiology, and End Results (SEER) registries in three regions of the United States: New Mexico, Los Angeles County, and Western Washington. Women aged 40–65 years were recruited in Western Washington; women aged 35 to 64 years were recruited in Los Angeles County; and women 18 years or older were recruited in New Mexico. We conducted an in-person interview (called the baseline assessment) within the first year (on average, 6 months) after a woman’s breast cancer diagnosis; the second assessment was conducted, on average, 30 months after a woman’s diagnosis (called the 30-month assessment) via in-person interview or self-administered questionnaire; and the third assessment was administered, on average, 40 months after a woman’s diagnosis (called the 40-month assessment) by telephone interview or mailed questionnaire in New Mexico, by mailed questionnaire plus telephone follow-up in Western Washington, and by telephone interview in Los Angeles County. All study participants provided informed consent at each assessment.

**Figure 1:**
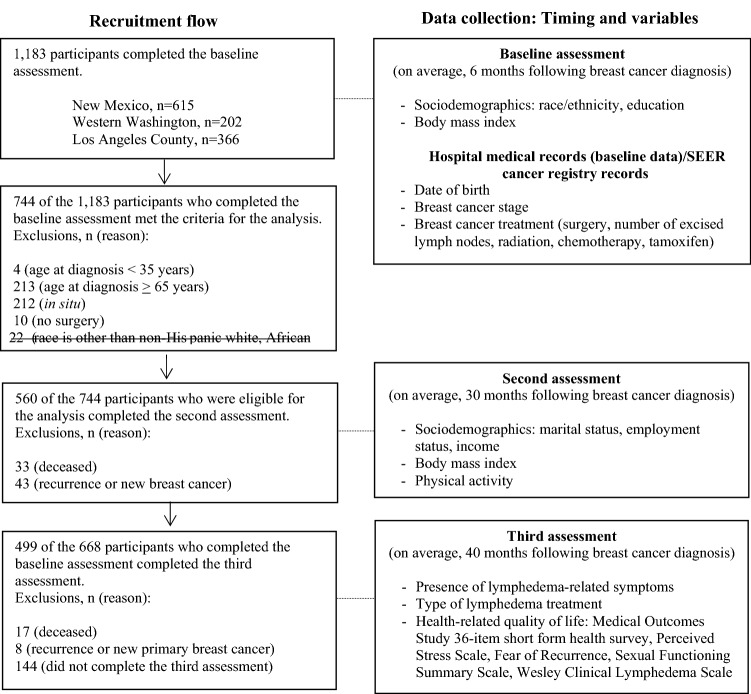
Flowchart describing recruitment flow and data collection. Out of 1183 participants who completed the baseline assessment, 499 were included in this study after exclusions.

For this analysis, we excluded 217 women who were younger than 35 years (n = 4) or older than 64 years (n = 213) at diagnosis in order to provide comparable age distributions across the three study sites. We also excluded 212 women who had in situ disease; 10 women who did not receive any type of surgery, which reduces risk of developing lymphedema; 101 women who had recurrence or new primary disease or who died before the 40-month assessment; and 144 women who did not complete the 40-month assessment. The final analytic cohort consisted of 499 women aged 35–64 years at diagnosis (Fig. 1).

### Data collection

Information on breast cancer diagnosis date, age at diagnosis, stage of cancer (SEER staging), treatment type(s), number of excised lymph nodes, and comorbid medical conditions was abstracted from SEER cancer registry records and hospital medical records. Using the information on comorbid medical conditions, we calculated Charlson Comorbidity Index^18,19^. Race/ethnicity, educational status, and height were self-reported by participants during the baseline assessment. Weight was collected as part of the 30-month assessment. We then calculated body mass index (BMI, weight (kg)/height (m^2^)). The Modifiable Activity Questionnaire, which has been validated previously^20^, was part of the 30-month assessment and was used to measure levels of sports and recreational physical activity. We calculated metabolic equivalents of task (MET) hours per week of sports and recreational physical activity^21^. Income, marital status, and employment were also self-reported in the 30-month assessment. The presence of lymphedema, lymphedema symptoms, and HRQoL information (perceived physical and mental health, stress, fear of cancer recurrence, sexual health, and lymphedema-specific HRQoL) were collected during the 40-month assessment.

#### Lymphedema and lymphedema-related symptoms

We defined lymphedema for participants using the following: “Sometimes the arm on the side on which you had breast cancer becomes swollen because of an accumulation of fluid in your arm. This is called lymphedema. Please do not confuse this with the temporary swelling that occurs after surgery.” Then women were asked whether, at any time since their breast cancer diagnosis they had experienced lymphedema in their arm on the side where breast cancer occurred (no, yes). Women who responded “yes,” were then asked: 1) when did they first experience lymphedema symptoms (month and year); 2) was lymphedema present at the time of assessment (no, yes); 3) did they receive any treatment for lymphedema (no, yes); and 4) did they have to change their clothing size or style because of problems associated with lymphedema (no, yes).

Women who reported lymphedema were queried about nine specific symptoms in their arm(s) either at the initial occurrence of lymphedema or at a later time: burning pain, numbness, feeling of tightness, feeling of tension, feeling of heaviness, feeling of hardness, loss of mobility, raised temperature (warmth)/redness, and dryness of skin. A symptom score, the total number of symptoms, was created for each woman who experienced lymphedema prior to or at the 40-month assessment. In the analysis, women who self-reported they experienced any of the nine specific lymphedema symptoms but were not experiencing lymphedema at the time of 40-month assessment were considered symptom-free (symptom score = 0).

#### Perceived physical and mental health

To assess women’s perceived physical and mental health, we used the Medical Outcomes Study 36-item short form health survey (SF-36)^22^. This widely-used measure assesses eight health concepts, each of which is a subscale of SF-36: (1) limitations in physical activities due to health problems (physical functioning); (2) limitations in usual role activities due to physical health problems (role-physical); (3) bodily pain; (4) general health perceptions (general health); (5) energy and fatigue (vitality); (6) limitations in social activities due to physical or emotional problems (social functioning); (7) limitations in usual role activities due to emotional problems (role-emotional); and (8) psychological distress and well-being (mental health). Raw scores on the SF-36 subscales were converted to z-scores and then converted to norm-based scores (with a standardized mean = 50, standard deviation = 10) using the 1998 general US population distribution to standardize the z-scores. The eight subscales were used to calculate a physical health component summary (PCS) score and a mental health component summary (MCS) score. The values for the subscales and the two summary scores range from 0 to 100 with higher scores indicating better functioning and health.

#### Perceived stress

A four-item version of the Perceived Stress Scale was used to measure the degree to which situations in one’s life are appraised as stressful^23^. Each item was rated on a 5-point Likert response scale and all four items were summed to obtain a total score ranging from 0 to 16 with higher score representing greater degrees of stress.

#### Fear of recurrence

We used a 5-item version of the Fear of Recurrence Scale^24,25^ which queried how much participants agreed or disagreed with statements that expressed concerns about future health status. For example, two of the statements were “I would like to feel more certain about my health” and “I worry that my cancer will return.” Women rated their responses on a Likert scale with values ranging from 1 (strongly disagree) to 5 (strongly agree). The total score on the Fear of Recurrence scale ranged from 5 to 25 with a higher score suggesting a greater fear of recurrence.

#### Sexual functioning

To measure sexual health after diagnosis, we used the Cancer Rehabilitation Evaluation System’s Sexual Functioning Summary Scale^26^. Women who were sexually active during the six months before the interview rated “Lack of a sexual interest”, “Difficultly in becoming sexually aroused”, “Unable to relax and enjoy sex”, and “Difficulty in having an orgasm” on a 4-point Likert response scale ranging from 0 (not a problem) to 3 (a serious problem). The Likert values were summed into a score that ranged from 0 to 12 with higher scores indicating greater difficulty with sexual functioning.

#### Lymphedema-specific HRQoL

Wesley Clinic Lymphedema Scale^27^ was used to measure lymphedema-specific HRQoL among women reporting lymphedema. Five items from the Functional Living Index-Cancer (FLIC)^28^ were selected and the term “cancer” or “illness” in these questions was replaced with the term “lymphedema” thereby providing a measure of lymphedema-specific HRQoL (e.g., “How much time do you spend thinking about your lymphedema in particular?”, “To what degree has your lymphedema imposed hardship on you (personally) in the past two weeks?”). Each question was scored on a 1 to 7 (e.g., “Constantly,” “Tremendous hardship”) scale, and items were summed to give a score. Total scores were divided by the possible maximum score to calculate the percent of maximum score. Higher scores indicated better HRQoL.

### Statistical methods

We used Pearson chi-square tests to compare participants’ characteristics across the three groups: 362 women who reported no lymphedema, 39 who had lymphedema in the past and were lymphedema symptom-free at the time of assessment and 98 who reported current lymphedema. One of the 98 women reported none of the lymphedema-related symptoms we queried and was considered lymphedema symptom-free in the following analyses. Analysis of covariance methods were used to compare each HRQoL measure across the aforementioned three groups, followed by Scheffé's post-hoc pair-wise comparisons^29^. PCS, MCS, and perceived stress were compared using standard analysis of variance. For other HRQoL measures, we used rank-based analysis of covariance methods because the distributions violated the normality assumption.

The participants’ characteristics that differed among the three groups based on the Pearson chi-square tests were considered for inclusion in analysis of covariance models. However, we did not include disease stage or lymphedema risk factors identified in our prior publication^30^ because disease stage is strongly associated with lymphedema risk factors and these risk factors are in the causal pathway, preceding the occurrence of lymphedema symptoms. BMI at the 30-month assessment also was not included in the model because of its high correlation with pre-diagnosis BMI which was also found to be a risk factor for lymphedema^30^ (Pearson correlation coefficient = 0.86). Since race/ethnicity was highly dependent on study site, i.e., all African American women were recruited in Los Angeles, all Hispanic women except one from Western Washington were recruited in New Mexico, non-Hispanic white women were recruited in either New Mexico or Seattle, we did not adjust for study site in addition to race/ethnicity. Thus, the statistical models used to assess associations between lymphedema and HRQoL among the three groups of women included only two design variables, age at the 40-month assessment and race/ethnicity.

We conducted tests for trend among women with lymphedema (n = 137) to assess whether HRQoL measures increased or decreased in a monotonic manner as the lymphedema symptom score (a continuous scale) increased. Furthermore, we examined whether any of the nine lymphedema symptoms was associated with any of the HRQoL measures by fitting analysis of covariance models. First, we applied a base model adjusted for age at the 40-month assessment and race/ethnicity. Then, we added “change in clothing size/style due to lymphedema” variable to the model along with any of the lymphedema symptoms that were found to be associated with HRQoL in the base model (*P* < 0.05). Additionally adjusting for surgery type, for which a statistically significant difference was detected between women who were lymphdema symptom-free (n = 40) and women with one or more lymphedema symptoms (n = 97) (*P* < 0.05), did not change the results substantially.

All analyses were performed using SAS software (SAS version 9.4; SAS Institute Inc, Cary, NC).

### Ethics approval

The HEAL Study was approved by the institutional review boards of the University of Southern California, University of New Mexico, Fred Hutchinson Cancer Research Center, University of Louisville, and Beckman Research Institute of City of Hope, according to assurances filed with and approved by the U.S. Department of Health and Human Services. All study methods were performed in accordance with the relevant guidelines and regulations.

## Results

Participants in the study belonged to one of four racial/ethnic groups: 272 (55%) from the New Mexico and Western Washington study sites were non-Hispanic whites; 155 (31%) from the Los Angeles County study site were African American; 54 (11%) from the New Mexico and Western Washington study sites were Hispanic; 18 (4%) from the New Mexico and Western Washington study sites were American Indian, Asian Pacific, or other races/ethnicities. Most women (71%, n = 352) had localized breast disease. A greater proportion of African American women (30%) reported at least one lymphedema symptom than non-Hispanic white women (15%), Hispanic women (15%), or American Indian/Asian Pacific women (6%) (*P* = 0.0002, Table 1). Furthermore, women were more likely to report at least one lymphedema symptom if they were younger, received more extensive surgery or chemotherapy, had 10 or more excised lymph nodes, or had BMI ≥ 30 kg/m^2^ (all *P*-values < 0.05). In addition, statistically significant differences in the distributions of race/ethnicity and surgery type were observed between lymphedema symptom-free women and women with lymphedema symptom(s) (both *P*-values < 0.05), but no associations were observed for other characteristics (all *P-*values > 0.05).

**Table 1:** Characteristics of 499 HEAL women.

Characteristics	All women (N = 499)	No lymphedema (N = 362)	Lymphedema symptom-free (N = 40)^a^	1 + lymphedema symptoms (N = 97)	*P-*value^b^
	N (%)	N (%)	N (%)	N (%)	
**Race/ethnicity**					
Non-Hispanic White	272 (55)	207 (76)	23 (8)	42 (15)	
African American	155 (31)	103 (66)	6 (4)	46 (30)	
Hispanic	54 (11)	36 (67)	10 (19)	8 (15)	
American Indian, Asian Pacific, other	18 (4)	16 (89)	1 (6)	1 (6)	0.0002^c^
**Age at 40-month assessment (years)**					
38–49	128 (26)	83 (65)	8 (6)	37 (29)	
50–59	215 (43)	156 (73)	19 (9)	40 (19)	
60–69	156 (31)	123 (79)	13 (8)	20 (13)	0.02
**Disease stage**					
Localized	352 (71)	264 (75)	29 (8)	59 (17)	
Regional	147 (29)	98 (67)	11 (7)	38 (26)	0.06
**Surgery type**					
Less than total mastectomy	320 (64)	243 (76)	29 (9)	48 (15)	
Total or modified radical mastectomy	179 (36)	119 (66)	11 (6)	49 (27)	0.003^c^
**Number of excised lymph nodes**					
0–9	158 (32)	136 (86)	7 (4)	15 (9)	
10–19	260 (52)	172 (66)	24 (9)	64 (25)	
≥ 20	81 (16)	54 (67)	9 (11)	18 (22)	0.0002
**Radiation therapy**					
No	172 (34)	120 (70)	13 (8)	39 (23)	
Yes	327 (66)	242 (74)	27 (8)	58 (18)	0.41
**Chemotherapy**					
No	260 (52)	203 (78)	21 (8)	36 (14)	
Yes	239 (48)	159 (67)	19 (8)	61 (26)	0.004
**Tamoxifen**					
No	178 (36)	130 (73)	14 (8)	34 (19)	
Yes	321 (64)	232 (72)	26 (8)	63 (20)	0.98
**Marital status at 30-month assessment**					
Married or living with a partner	291 (58)	218 (75)	22 (8)	51 (18)	
Widowed/divorced/separated	164 (33)	114 (70)	14 (9)	36 (22)	
Never married	34 (7)	24 (71)	3 (9)	7 (21)	0.79
Missing	10 (2)	6 (60)	1 (10)	3 (30)	
**Education**					
High school or less	128 (26)	94 (73)	11 (9)	23 (18)	
Some college	181 (36)	129 (71)	14 (8)	38 (21)	
College graduate	94 (19)	63 (67)	5 (5)	26 (28)	0.11
Graduate studies	96 (19)	76 (79)	10 (10)	10 (10)	
**Employment status at 30-month assessment**					
Currently working	323 (65)	236 (73)	30 (9)	57 (18)	
Unemployed/not working/ retired/disabled	165 (33)	120 (73)	9 (5)	36 (22)	0.22
Missing	11 (2)	6 (55)	1 (9)	4 (36)	
**Income at 30-month assessment (US dollars)**					
≤ 50,000	245 (49)	179 (73)	18 (7)	48 (20)	
> 50,000	214 (43)	153 (72)	19 (9)	42 (20)	0.83
Missing	40 (8)	30 (75)	3 (8)	7 (18)	
**Physical activity at 30-month assessment (MET hours)**					
0	69 (14)	54 (78)	3 (4)	12 (17)	
0.1–8.9	209 (42)	153 (73)	14 (7)	42 (20)	
≥ 9	209 (42)	147 (70)	22 (11)	40 (19)	0.42
Missing	12 (2)	8 (67)	1 (8)	3 (25)	
**Body mass index at 30-month assessment (kg/m**^**2**^**)**					
< 25	173 (35)	139 (80)	8 (5)	26 (15)	
25–29.9	134 (27)	98 (73)	15 (11)	21 (16)	
≥ 30 or above	153 (31)	98 (64)	11 (7)	44 (29)	0.002
Missing	39 (8)	27 (69)	6 (15)	6 (15)	
**Charlson Comorbidity Index**					
0	445 (89)	321 (72)	38 (9)	86 (19)	
≥ 1	53 (11)	40 (75)	2 (4)	11 (21)	0.48
Missing	1 (0)	1 (100)	0 (0)	0 (0)	
**Lymphedema treatment**^d^					
Never	62 (45)	–	22 (35)	40 (65)	
Ever	75 (55)	–	18 (24)	57 (76)	0.14
**Change in clothing size/style due to lymphedema**^d^					
No	99 (72)	–	33 (33)	66 (67)	
Yes	38 (28)	–	7 (18)	31 (82)	0.09

*MET* metabolic equivalents of energy expenditure, *N* number.

^a^One of the 98 women reporting current lymphedema at the 40-month assessment did not report any of the lymphedema-related symptoms we queried; this woman is included in the “Lymphedema symptom-free” group.

^b^*P*-values are based on Chi-square test without missing values.

^c^A statistically significant difference was detected based on Chi-square test comparing between women who were lymphedema symptom-free and women with 1 or more lymphedema symptoms (*P* < 0.05).

^d^The proportions are based on 137 women who reported past or current lymphedema.

Among the 137 (28%) women who reported lymphedema, more than half (55%) had received lymphedema treatment and 98 (72%) were experiencing lymphedema at the time of the 40-month assessment. Among the 97 women reporting current lymphedema with at least one lymphedema symptom, the mean number of lymphedema symptoms reported by these women was 4.0 (standard deviation = 1.8), and 32% reported they had to change clothing size or style due to lymphedema. The most common self-reported symptoms were feeling heaviness (52%), numbness (47%), and tightness (45%).

### Lymphedema and HRQoL measures

**Figure 2:**
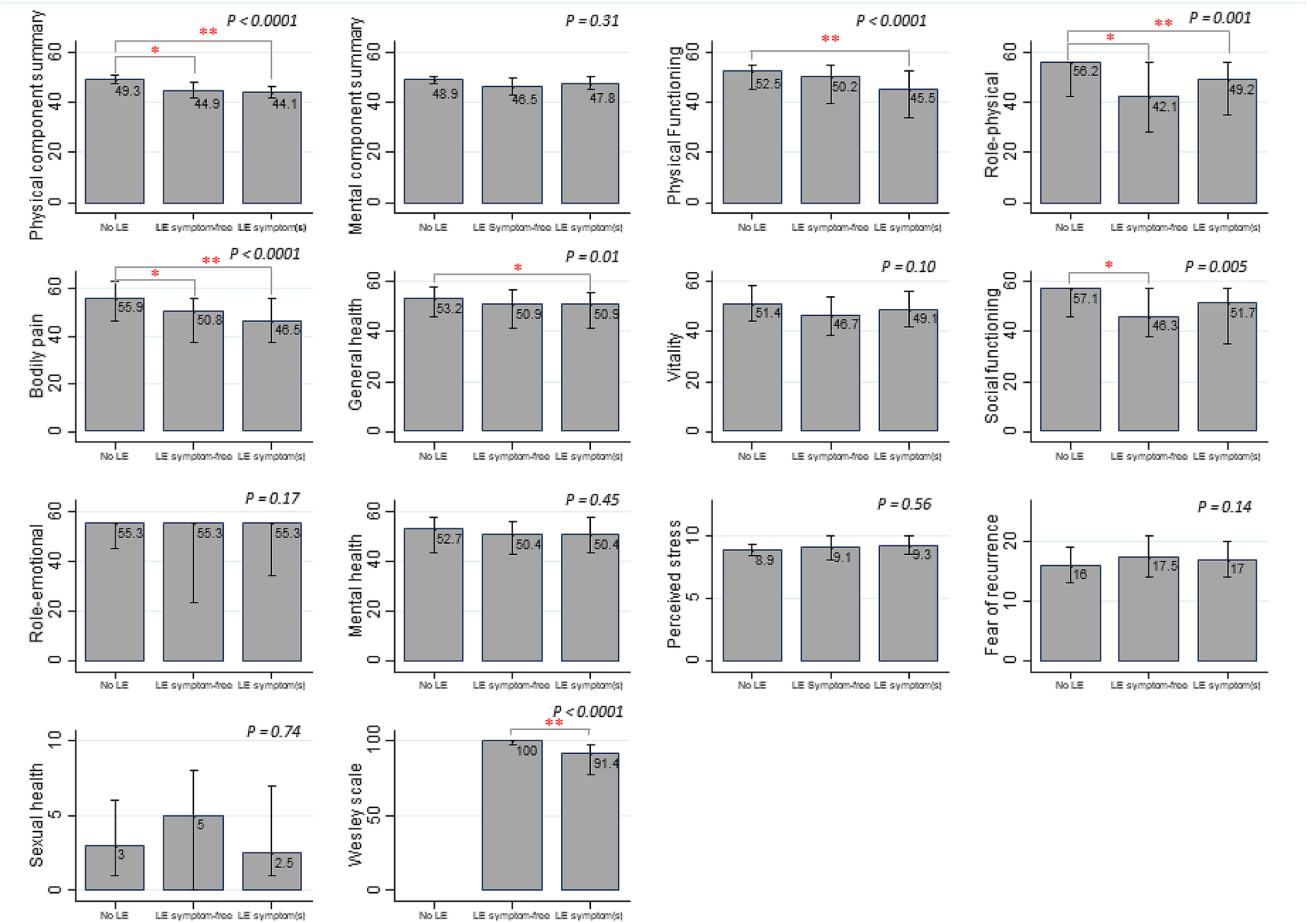
Lymphedema and health-related quality of life measures. Adjusted means are presented for the SF-36 physical and mental component summary scores and perceived stress scale. Medians are presented for the other health-related quality of life measures. The vertical lines represent the 95% confidence intervals for the means and the interquartile ranges for the medians. A higher score indicates better health-related quality of life in Medical outcomes study 36-item short form health survey scales and Wesley Clinical Lymphedema Scale and worse health-related quality of life in perceived sterss, fear of recurrence, and sexual health scales. *P* values are based on F statistics from the analysis of covariance in 499 women (for the analysis of sexual health, only 288 women who reported being sexually active during the 6 months prior to 40-month assessment were included). Asterisk(s) indicate a statistically significant difference observed in Scheffé's pair-wise multiple comparison test (*0.01 ≤ *P* value < 0.05, **P value < 0.01). *LE* lymphedema.

When we compared HRQoL measures across the three groups of women (no lymphedema, lymphedema without lymphedema symptom, and lymphedema with lymphedema symptom(s)), we observed statistically significant differences between the groups for PCS score and the following SF-36 subscales: physical functioning, role-physical, bodily pain, general health, and social functioning (all *P-*values ≤ 0.01), but not for other HRQoL measures (Fig. 2, Supplementary Table S1). Of those scales, the adjusted mean score in women reporting at least one lymphedema-related symptom was below 50 (general population mean) for PCS (44.1), physical functioning (45.5), role-physical (49.2), and bodily pain (46.5). Post-hoc pair-wise comparisons showed that PCS score and SF-36 physical functioning, role-physical, bodily pain, general health, and social functioning subscale scores were worse for women who reported lymphedema with or without current lymphedema/lymphedema symptom(s) than for women without lymphedema (all *P*-values < 0.05). The differences in SF-36 physical functioning and general health subscales only existed between women without lymphedema and those who reported at least one lymphedema symptom (*P-*values ≤ 0.03). Lymphedema-specific HRQoL was lower in women with at least one lymphedema symptom than lymphedema symptom-free women (*P*-value < 0.0001).

### Lymphedema symptom score and HRQoL measures

The analysis restricted to the 137 women who reported lymphedema showed that the lymphedema symptom score was inversely associated with lymphedema-specific HRQoL, PCS score, and SF-36 physical functioning subscale, and was positively associated with perceived stress (all *P*-values for trend ≤ 0.01, Supplementary Table S1).

### Lymphedema symptoms and perceived physical and mental health

Using the base model (adjusted for age at the 40-month assessment and race/ethnicity), we found that the following lymphedema symptoms were associated with lower PCS in the 137 women who reported lymphedema: numbness, tension, hardness, and warmth/redness (all *P-*values ≤ 0.03, Table 2). The analysis, in which we mutually adjusted for these four symptoms and change in clothing size/style, showed that only tension was independently associated with lower PCS (*P-*value = 0.01). In contrast, we found no association between MCS and the presence of any lymphedema symptom (all *P-*value ≥ 0.18).

**Table 2:** Lymphedema symptoms and physical and mental component summary scores.

Lymphedema symptom	N	*β* (SE)^a^	Mean^a^	*P*-value^a^	*β* (SE)^a,b^	*P*-value^a,b^
**SF-36 PCS**						
**Burning pain**						
No	115		43.29			
Yes	22	− 2.66 (2.36)	40.63	0.26	–	–
**Numbness**						
No	73		44.83			
Yes	64	− 3.99 (1.75)	40.84	0.02	− 1.89 (1.86)	0.31
**Tightness**						
No	75		43.97			
Yes	62	− 2.13 (1.75)	41.84	0.23	–	–
**Tension**						
No	102		44.15			
Yes	35	− 6.47 (1.91)	37.68	0.001	− 5.16 (1.99)	0.01
**Heaviness**						
No	66		44.47			
Yes	71	− 3.00 (1.80)	41.47	0.10	–	–
**Hardness**						
No	106		43.77			
Yes	31	− 4.47 (2.02)	39.30	0.03	− 2.18 (2.08)	0.30
**Loss of mobility**						
No	105		43.89			
Yes	32	− 3.02 (2.09)	40.87	0.15	–	–
**Warmth/redness**						
No	103		43.88			
Yes	34	− 5.04 (1.98)	38.84	0.01	− 1.91 (2.18)	0.38
**Dry skin**						
No	101		43.21			
Yes	36	− 0.96 (2.01)	42.25	0.63	–	–
**SF-36 MCS**						
**Burning pain**						
No	115		46.57			
Yes	22	− 3.46 (2.56)	43.11	0.18	–	–
**Numbness**						
No	73		47.00			
Yes	64	− 1.70 (1.94)	45.30	0.38	–	–
**Tightness**						
No	75		45.97			
Yes	62	0.60 (1.91)	46.57	0.75	–	–
**Tension**						
No	102		46.62			
Yes	35	− 2.21 (2.15)	44.40	0.31	–	–
**Heaviness**						
No	66		45.93			
Yes	71	0.64 (1.97)	46.57	0.75	–	–
**Hardness**						
No	106		46.33			
Yes	31	− 0.56 (2.24)	45.77	0.80	–	–
**Loss of mobility**						
No	105		47.01			
Yes	32	− 2.75 (2.3)	44.27	0.23	–	–
**Warmth/redness**						
No	103		46.62			
Yes	34	− 2.30 (2.20)	44.32	0.30	–	–
**Dry skin**						
No	101		46.71			
Yes	36	− 2.64 (2.17)	44.07	0.23	–	–

*MCS* mental component summary, *PCS* physical component summary, *SF-36* 36-item short form health survey, *N* number, *SE* standard error.

^a^Standard analysis of covariance model adjusting for age at 40-month assessment and race/ethnicity was used.

^b^Mutually adjusted for numbness, tension, hardness, and warmth/redness, and additionally adjusted for change in clothing size/style due to lymphedema.

### Lymphedema symptoms and perceived stress, fear of recurrence, and sexual functioning

The analysis using the base model showed that greater perceived stress was associated with lymphedema-related symptoms of numbness, tension, warmth/redness, or dryness of skin (all *P-*values < 0.05, Table 3). However, when these four symptoms and change in clothing size/style were all added to the base model, none of the association remained (all *P-*values ≥ 0.09). Furthermore, we assessed the associations of lymphedema symptoms with fear of recurrence and sexual functioning summary scales and found no association (data not shown).

**Table 3:** Lymphedema symptoms and perceived stress.

Lymphedema symptom	N	Perceived stress				
		*β* (SE)^a^	Mean^a^	*P*-value^a^	*β* (SE)^a,b^	*P*-value^a,b^
**Burning pain**						
No	115		9.14			
Yes	22	1.16 (0.74)	10.30	0.12	–	–
**Numbness**						
No	73		8.76			
Yes	64	1.10 (0.55)	9.86	0.049	0.46 (0.61)	0.45
**Tightness**						
No	75		9.17			
Yes	62	0.20 (0.55)	9.37	0.72	–	–
**Tension**						
No	102		9.01			
Yes	35	1.45 (0.61)	10.46	0.02	1.08 (0.64)	0.09
**Heaviness**						
No	66		9.02			
Yes	71	0.48 (0.57)	9.51	0.40	–	–
**Hardness**						
No	106		9.26			
Yes	31	− 0.02 (0.6)	9.24	0.98	–	–
**Loss of mobility**						
No	105		8.97			
Yes	32	1.00 (0.66)	9.97	0.13	–	–
**Warmth/redness**						
No	103		9.03			
Yes	34	1.33 (0.63)	10.36	0.04	0.67 (0.70)	0.34
**Dry skin**						
No	101		9.03			
Yes	36	1.27 (0.62)	10.30	0.04	0.88 (0.65)	0.18

*N* number, *SE* standard error.

^a^Standard analysis of covariance model adjusting for age at 40-month assessment and race/ethnicity was used.

^b^Mutually adjusted for numbness, tension, warmth/redness, and dry skin, and additionally adjusted for change in clothing size/style due to lymphedema.

### Lymphedema symptoms and lymphedema-specific HRQoL

Using the base model, we found that all symptoms except for dryness of skin were associated with poorer lymphedema-specific HRQoL (all *P-*values ≤ 0.0002, Table 4). After mutual adjustment of these symptoms and change in clothing size/style, burning pain, tension, heaviness, and warmth/redness were independently associated with poorer lymphedema-specific HRQoL (all *P-*values < 0.05).

**Table 4:** Lymphedema symptoms and lymphedema-specific health-related quality of life.

Lymphedema symptom	Lymphedema-specific health-related quality of life			
	N	Median (IQR)	*P*-value^a^	*P*-value^a,b^
**Burning pain**				
No	115	94.29 (85.71–100)		
Yes	22	81.43 (51.43–91.43)	0.0002	0.02
**Numbness**				
No	73	97.14 (91.43–100)		
Yes	64	85.71 (65.71–94.29)	< 0.0001	0.26
**Tightness**				
No	75	97.14 (91.43–100)		
Yes	62	88.57 (62.86–94.29)	< 0.0001	0.08
**Tension**				
No	102	97.14 (88.57–100)		
Yes	35	82.86 (51.43–91.43)	< 0.0001	0.01
**Heaviness**				
No	66	100 (94.29–100)		
Yes	71	85.71 (68.57–94.29)	< 0.0001	0.02
**Hardness**				
No	106	97.14 (85.71–100)		
Yes	31	80 (60–94.29)	0.0002	0.73
**Loss of mobility**				
No	105	97.14 (88.57–100)		
Yes	32	85.71 (58.57–92.86)	< 0.0001	0.08
**Warmth/redness**				
No	103	97.14 (88.57–100)		
Yes	34	78.57 (54.29–91.43)	< 0.0001	0.049
**Dry skin**				
No	101	94.29 (85.71–100)		
Yes	36	88.57 (72.86–100)	0.15	–

*IQR* interquartile range, *N* number.

^a^Rank-based analysis of covariance model adjusting for age at 40-month assessment and race/ethnicity was used.

^b^Mutually adjusted for all symptoms excluding dry skin, and additionally adjusted for change in clothing size/style due to lymphedema.

## Discussion

In this multi-racial-ethnic cohort of breast cancer survivors, women experiencing lymphedema symptom(s) reported poorer HRQoL than women without lymphedema, particularly in the domains of physical functioning, role-physical, bodily pain, and general health. Lymphedema-specific HRQoL, perceived physical health, and perceived stress were particularly poorer among women reporting lymphedema symptoms of burning pain, tension, heaviness, or warmness/redness and those reporting a greater number of lymphedema-related symptoms.

Our study results are consistent with previous reports that demonstrated an association between breast cancer-related lymphedema and poorer HRQoL, specifically how self-rated physical health decreases as the number of self-reported lymphedema symptoms increases^6,8^. Of the nine symptoms we queried in the HEAL Study, tension in the arm was the only symptom that was independently associated with the SF-36 PCS after adjusting for age, race/ethnicity, other symptoms, and change in clothing size/style due to lymphedema. Otherwise, none of the lymphedema symptoms queried in our study or the total number of symptoms was associated with the SF-36 MCS. Our findings are consistent with the study conducted by Dawes et al. where an inverse association was found with physical health, and not with mental health^31^, but inconsistent with other studies that showed a negative psychosocial impact on individuals affected by lymphedema^32,33^. The lack of any association with mental health in the present study could be due to poorer discriminatory power of the SF-36 MCS for detecting the association of lymphedema with mental health compared to more specific instruments, such as FLIC^34^. In fact, in the HEAL study, the number of lymphedema symptoms a woman reported was inversely associated with her HRQoL as measured by Wesley Clinic Lymphedema Scale which is an adapted version of the FLIC questionnaire. Specifically, women reporting burning pain, tension, heaviness, or warmth/redness reported poorer HRQoL. These associations were independent of change in clothing size/style due to lymphedema, suggesting that lower values on the HRQoL scale may not be due solely to lymphedema-related increases in arm size.

Furthermore, our data also show that the scale of perceived stress increases as the number of self-reported lymphedema symptoms increases. Although we did not find any association between specific lymphedema symptoms and SF-36 MCS, the association with greater perceived stress suggests the importance of providing counseling and support to identify and address stressors that lymphedema-affected women are experiencing. Fear of cancer recurrence is an important source of distress after breast cancer treatment^35^, and some qualitative studies report that lymphedema-induced swelling or other symptoms can trigger fears about cancer recurrence^36^. However, our quantitative analysis did not demonstrate this association.

The sexuality among breast cancer survivors has received substantial research attention; however, few data exist on the association between breast cancer-related lymphedema and quality of sexual life. Previous studies have shown that breast cancer survivors with lymphedema had decreased sexual intimacy, desire, and activity^37–39^. However, in the present study, no association was found between lymphedema and being sexually active or between perceived sexual health and lymphedema symptoms (data not shown). Since sexuality was assessed at about 40 months after diagnosis in the HEAL cohort, we might have missed any change in a woman’s quality of sexual life that might have occurred prior to or after the 40-month assessment.

The strengths of this study include our comprehensive assessment of HRQoL and various symptoms of lymphedema, which allowed us to examine associations between different lymphedema symptoms and many HRQoL domains in breast cancer survivors. Our findings suggest areas that should be considered in efforts to improve the management of lymphedema and health-related quality of life after breast cancer therapy. Despite these strengths, the following limitations should be considered when interpreting our study results. In the HEAL Study, presence or absence of lymphedema or lymphedema symptoms is solely based on self-report. Although self-report of lymphedema has been shown to yield high sensitivity overall, specificity for mild cases (a difference of ≤ 2 cm) may be lower^40^, which may have resulted in classifying some women as having lymphedema when they in fact did not have lymphedema. Furthermore, we did not collect information on whether lymphedema occurred on the dominant arm. Women whose lymphedema was on the dominant arm might have experienced more limitations in arm mobility. Another limitation of the present study is its cross-sectional nature. Because HRQoL and lymphedema were measured at the same time, we cannot determine if HRQoL is a result of lymphedema or if it is independent and unrelated to lymphedema.

Overall, our findings support the conclusion that breast cancer survivors with greater number of lymphedema symptoms or specific symptoms, including burning pain, tension, heaviness, and warmth/redness have poorer HRQoL, particularly in the domains of perceived physical health and stress. As previous studies have recommended an assessment of lymphedema symptoms in the follow-up care of breast cancer survivors to detect mild lymphedema which may not be detected by arm size/volume measurements^41^, our results also underscore the importance of assessing specific lymphedema symptoms in addition to arm size/volume measurements in order to identify needs of the affected individuals and provide appropriate care, such as stress and pain management, thereby improving their HRQoL.

## Supplementary Information

Supplementary Table

## Abbreviations

BMI: Body mass index
FLIC: Functional living index-cancer
HEAL: Health, eating, activity and lifestyle
HRQoL: Health-related quality of life
kg: Kilogram
SF-36: Medical outcomes study 36-item short form health survey
MCS: Mental health component summary
MET: Metabolic equivalents of task
m: Meter
PCS: Physical health component summary
SEER: Surveillance, epidemiology, and end results
