# Supplementary material for: Self-reported symptoms of arm lymphedema and health-related quality of life among female breast cancer survivors
Source: Sci Rep. 2021 May 21;11:10701. doi: 10.1038/s41598-021-89055-0 (PMC8139966; doi:10.1038/s41598-021-89055-0)
Supplement: Supplementary file 1 — Supplementary Table [file 41598_2021_89055_MOESM1_ESM.pdf]

Supplementary Table. Lymphedema and health-related quality of life measures

Adjusted means and standard errors are presented for the SF-36 physical and mental component summary scores and perceived stress scale. Medians and interquartile ranges are presented for the other health-related quality of life measures. *P* values for F statistics from the analysis of covariance in 499 women and *P* values from the analysis of symptom score in 137 women who self-reported lymphedema are presented.

**Quality of life measure (score range)[a]**	**No lymphedema Adjusted mean (SE)/ Median (IQR)**	**Lymphedema symptom-free Adjusted mean (SE)/ Median (IQR)**	**1+ lymphedema symptoms Adjusted mean (SE)/ Median (IQR))**	***P*-value**	***P*-value for trend test [d]**
	N=362	N=40	N=97		
MOS SF-36 PCS[b] (0-100)	49.3 (0.8)	44.9 (1.7)[h]	44.1 (1.2)[g]	*<0.0001*	*0.001*
MOS SF-36 MCS[b] (0-100)	48.9 (0.8)	46.5 (1.7)	47.8 (1.2)	*0.31*	*0.24*
MOS SF-36 Subscales[c] (0-100)					
Physical Functioning	52.5 (45.5-54.8)	50.2 (39.7-54.8)	45.5 (33.8-52.5)[g]	*<0.0001*	*<0.0001*
Role-Physical	56.2 (42.1-56.2)	42.1 (28.0-56.2)[h]	49.2 (35.0-56.2)[g]	*0.001*	*0.07*
Bodily Pain	55.9 (46.5-62.7)	50.8 (37.5-55.9)[h]	46.5 (37.5-55.9)[g]	*<0.0001*	*0.07*
General Health	53.2 (46.2-57.9)	50.9 (41.5-56.7)	50.9 (41.5-55.6)[g]	*0.01*	*0.07*
Vitality	51.4 (44.3-58.5)	46.7 (38.4-53.8)	49.1 (42.0-56.2)	*0.10*	*0.30*
Social Functioning	57.1 (46.3-57.1)	46.3 (38.1-57.1)[h]	51.7 (35.4-57.1)	*0.005*	*0.23*
Role-Emotional	55.3 (44.8-55.3)	55.3 (23.7-55.3)	55.3 (34.3-55.3)	*0.17*	*0.31*
Mental Health	52.7 (43.6-57.3)	50.4 (42.5-56.1)	50.4 (43.6-57.3)	*0.45*	*0.75*
Perceived Stress Scale[b,c] (0-16)	8.9 (0.2)	9.1 (0.5)	9.3 (0.4)	*0.56*	*0.01*
Fear of Recurrence Scale[d] (5-25)	16.0 (13.0-19.0)	17.5 (14.0-21.0)	17.0 (14.0-20.0)	*0.14*	*0.79*
Wesley Clinical Lymphedema Scale[e,f] (0-100)	-	100 (97.1-100)	91.4 (77.1-97.1)	*<0.0001*	*<0.0001*
	N=207	N=25	N=56		
Sexual Functioning Summary Scale[c,e] (0-12)	3.0 (1.0-6.0)	5.0 (0.0-8.0)	2.5 (1.0-7.0)	*0.74*	*0.43*

Abbreviation. IQR: interquartile range; MCS: mental component summary; MOS SF-36: Medical outcomes study 36-item short form health survey; N: number; PCS: physical component summary; SE: standard error

[a] A higher score indicates better health-related quality of life in MOS scales and Wesley Clinical Lymphedema Scale and worse health-related quality of life in other scales.

[b] Standard analysis of covariance model adjusting for age at 40-month assessment and race/ethnicity was used.

[c] A higher score indicates greater perceived stress.

[d] Rank-based analysis of covariance model adjusting for age at 40-month assessment and race/ethnicity was used.

[e] Only 137 women who reported lymphedema were included in the analysis of symptom score adjusting for age at 40-month assessment and race/ethnicity.

[f] Only 288 women who reported being sexually active during the past 6 months prior to 40-month assessment were included.

[g] Scheffé's pair-wise multiple comparison test detected a statistically significant difference between women without lymphedema and women with at least one symptom.

[h] Scheffé's pair-wise multiple comparison test detected a statistically significant difference between women without lymphedema and women with lymphedema and no symptom.
